# Supervised Clustering Based on DPClusO: Prediction of Plant-Disease Relations Using Jamu Formulas of KNApSAcK Database

**DOI:** 10.1155/2014/831751

**Published:** 2014-04-07

**Authors:** Sony Hartono Wijaya, Husnawati Husnawati, Farit Mochamad Afendi, Irmanida Batubara, Latifah K. Darusman, Md. Altaf-Ul-Amin, Tetsuo Sato, Naoaki Ono, Tadao Sugiura, Shigehiko Kanaya

**Affiliations:** ^1^Graduate School of Information Science, Nara Institute of Science and Technology, 8916-5 Takayama, Ikoma, Nara 630-0192, Japan; ^2^Department of Computer Science, Bogor Agricultural University, Kampus IPB Dramaga, Jl. Meranti, Bogor 16680, Indonesia; ^3^Department of Biochemistry, Bogor Agricultural University, Kampus IPB Dramaga, Jl. Meranti, Bogor 16680, Indonesia; ^4^Department of Statistics, Bogor Agricultural University, Kampus IPB Dramaga, Jl. Meranti, Bogor 16680, Indonesia; ^5^Biopharmaca Research Center, Bogor Agricultural University, Kampus IPB Taman Kencana, Jl. Taman Kencana No. 3, Bogor 16151, Indonesia

## Abstract

Indonesia has the largest medicinal plant species in the world and these plants are used as Jamu medicines. Jamu medicines are popular traditional medicines from Indonesia and we need to systemize the formulation of Jamu and develop basic scientific principles of Jamu to meet the requirement of Indonesian Healthcare System. We propose a new approach to predict the relation between plant and disease using network analysis and supervised clustering. At the preliminary step, we assigned 3138 Jamu formulas to 116 diseases of International Classification of Diseases (ver. 10) which belong to 18 classes of disease from National Center for Biotechnology Information. The correlation measures between Jamu pairs were determined based on their ingredient similarity. Networks are constructed and analyzed by selecting highly correlated Jamu pairs. Clusters were then generated by using the network clustering algorithm DPClusO. By using matching score of a cluster, the dominant disease and high frequency plant associated to the cluster are determined. The plant to disease relations predicted by our method were evaluated in the context of previously published results and were found to produce around 90% successful predictions.

## 1. Introduction

Big data biology, which is a discipline of data-intensive science, has emerged because of the rapid increasing of data in omics fields such as genomics, transcriptomics, proteomics, and metabolomics as well as in several other fields such as ethnomedicinal survey. The number of medicinal plants is estimated to be 40,000 to 70,000 around the world [1] and many countries utilize these plants as blended herbal medicines, for example, China (traditional Chinese medicine), Japan (Kampo medicine), India (Ayurveda, Siddha, and Unani), and Indonesia (Jamu). Nowadays, the use of traditional medicines is rapidly increasing [2, 3]. These medicines consist of ingredients made from plants, animals, minerals, or combination of them. The traditional medicines have been used for generations for treatments of diseases or maintaining health of people and the most popular form of traditional medicine is herbal medicine. Blended herbal medicines as well as single herb medicines include a large number of constituent substances which exert effects on human physiology through a variety of biological pathways. The KNApSAcK Family database systems can be used to comprehensively understand the medicinal usage of plants based upon traditional and modern knowledge [4, 5]. This database has information about the selected herbal ingredients, that is, the formulas of Kampo and Jamu, omics information of plants and humans, and physiological activities in humans. Jamu is generally composed based on the experience of the users for decades or even hundreds of years. However, versatile scientific analyses are needed to support their efficacy and their safety. Attaining this objective is in accordance with the 2010 policy of the Ministry of Health of Indonesian Government about scientification of Jamu. Thus, it is required to systemize the formulations and develop basic scientific principles of Jamu to meet the requirement of Indonesian Healthcare System. Afendi et al. initiated and conducted scientific analysis of Jamu for finding the correlation between plants, Jamu, and their efficacy using statistical methods [6–8]. They used Biplot, partial least squares (PLS), and bootstrapping methods to summarize the data and also focused on prediction of Jamu formulations. These methods give a good understanding about relationship between plants, Jamu, and their efficacy. Among 465 plants used in 3138 Jamu, 190 plants were shown to be effective for at least one efficacy and these plants were considered to be the main ingredients of Jamu. The other 275 plants are considered to be supporting ingredients in Jamu because their efficacy has not been established yet.

Network biology can be defined as the study of the network representations of molecular interactions, both to analyze such networks and to use them as a tool to make biological predictions [9]. This study includes modelling, analysis, and visualizations, which holds important task in life science today [10]. Network analysis has been increasingly utilized in interpreting high throughput data on omics information, including transcriptional regulatory networks [11], coexpression networks [12], and protein-protein interactions [13]. We can easily describe relationship between entities in the network and also concentrate on part of the network consisting of important nodes or edges. These advantages can be adopted for analyzing medicinal usage of plants in Jamu and diseases. Network analysis provides information about groups of Jamu that are closely related to each other in terms of ingredient similarity and thus allows precise investigation to relate plants to diseases. On the other hand, multivariate statistical methods such as PLS can assign plants to efficacy by global linear modeling of the Jamu ingredients and efficacy. However, there is still lack of appropriate network based methods to learn how and why many plants are grouped in certain Jamu formula and the combination rule embedding numerous Jamu formulas.

It is needed to explore the relationship between Indonesian herbal plants used in Jamu medicines and the diseases which are treated using Jamu medicines. When effectiveness of a plant against a disease is firmly established, then further analysis about that plant can be proceeded to molecular level to pinpoint the drug targets. The present study developed a network based approach for prediction of plant-disease relations. We utilized the Jamu data from the KNApSAcK database. A Jamu network was constructed based on the similarity of their ingredients and then Jamu clusters were generated using the network clustering algorithm DPClusO [14, 15]. Plant-disease relations were then predicted by determining the dominant diseases and plants associated with selected Jamu clusters.

## 2. Methods

### 2.1. Concept of the Methodology

**Figure 1:**
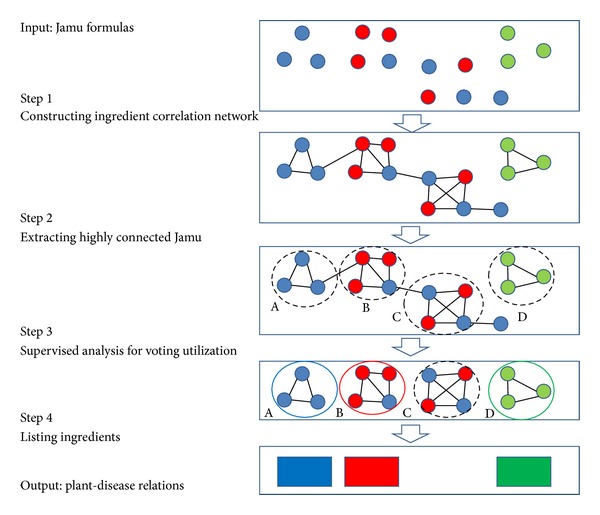
Concept of the methodology: network construction based on ingredient similarity between individual Jamu medicines, network clustering, and classification of medicinal plants to dominant disease.

Jamu medicines consist of combination of medicinal plants and are used to treat versatile diseases. In this work we exploit the ingredient similarity between Jamu medicines to predict plant-disease relations. The concept of the proposed method is depicted in Figure 1. In step 1 a network is constructed where a node is a Jamu medicine and an edge represents high ingredient similarity between the corresponding Jamu pair. In Figure 1, the nodes of the same color indicate the Jamu medicines used for the same disease. The similarity is represented by Pearson correlation coefficient [16, 17]; that is,

(1) $$\begin{array}{c} \text{corr}\left(X,Y\right)=\frac{\sum_{i=1}^{l}\left(x_{i}-\bar{x}\right)\left(y_{i}-\bar{y}\right)}{\sqrt{{\sum_{i=1}^{l}\left(x_{i}-\bar{x}\right)}^{2}{\sum_{i=1}^{l}\left(y_{i}-\bar{y}\right)}^{2}}}, \end{array}$$

where *x*_*i*_ is the weight of plant-*i* in Jamu *X*, *y*_*i*_ is the weight of plant-*i* in Jamu *Y*, $\bar{x}$ is mean of Jamu *X*, and $\bar{y}$ is mean of Jamu *Y*. The higher similarity between Jamu pairs the higher the correlation value. In the present study, *x*_*i*_ and *y*_*i*_ are assigned as 1 or 0 in cases the *i*th plant is, respectively, included or not included in the formula. Under such condition, Pearson correlation corresponds to fourfold point correlation coefficient; that is,

(2) $$\begin{array}{c} \text{corr}\left(X,Y\right)=\;\frac{ad-bc}{\sqrt{\left(a+b\right)\left(a+c\right)\left(b+d\right)\left(c+d\right)}}, \end{array}$$

where *a*, *b*, *c*, and *d* represent the numbers of plants included in both *X* and *Y*, in only *X*, in only *Y*, and in neither *X* nor *Y*, respectively.

In step 2 the Jamu clusters are generated using network clustering algorithm DPClusO. DPClusO can generate clusters characterized by high density and identified by periphery; that is, the Jamu medicines belonging to a cluster are highly cohesive and separated by a natural boundary. Such clusters contain potential information about plant-disease relations.

In step 3 we assess disease-dominant clusters based on matching score represented by the following equation:

(3) matching  score =number  of  
Jamu
  belonging  to  the  same  diseasetotal  number  of  
Jamu
  in  the  cluster.

Matching score of a cluster is the ratio of the highest number of Jamu associated with a single disease to the total number of Jamu in the cluster. We assign a disease to a cluster for which the matching score is greater than a threshold value. In step 4, we determine the frequency of plants associated with a cluster if and only if a disease is assigned to it in the previous step. The highest frequency plant associated to a cluster is considered to be related to the disease assigned to that cluster. True positive rates (TPR) or sensitivity was used to evaluate resulting plants. TPR is the proportion of the true positive predictions out of all the true predictions, defined by the following formula [18]:

(4) $$\begin{array}{c} \text{TPR}=\frac{\text{TP}}{\text{TP}+\text{FN}}, \end{array}$$

where true positive (TP) is the number of correctly classified and false negative (FN) is the number of incorrectly rejected entities. We refer to the proposed method as supervised clustering because after generation of the clusters we narrow down the candidate clusters for further analysis based on supervised learning and thus improve the accuracy of prediction of the proposed method.

## 3. Result and Discussion 

### 3.1. Construction and Comparison of Jamu and Random Networks

We used the same number of Jamu formulas from previous research [6], 3138 Jamu formulas, and the set union of all formulas consists of 465 plants. We assigned 3138 Jamu formulas to 116 diseases of International Classification of Diseases (ICD) version 10 from World Health Organization (WHO, Table 1) [19]. Those 116 diseases are mapped to 18 classes of disease, which contains 16 classes of disease from National Center for Biotechnology Information (NCBI) [20] and 2 additional classes.  Table 2 shows distribution of 3138 Jamu into 18 classes of disease. According to this classification, most Jamu formulas are useful for relieving muscle and bone, nutritional and metabolic diseases, and the digestive system. Furthermore, there is no Jamu formula classified into glands and hormones and neonatal disease classes. We excluded 4 Jamu formulas which are used to treat fever in the evaluation process because this symptom is very general and almost appeared in all disease classes. Jamu-plant-disease relations can be represented using 2 matrices: first matrix is Jamu-plant relation with dimension 3138 × 465 and the second matrix is Jamu-disease relation with dimension 3138 × 18.

**Figure 2:**
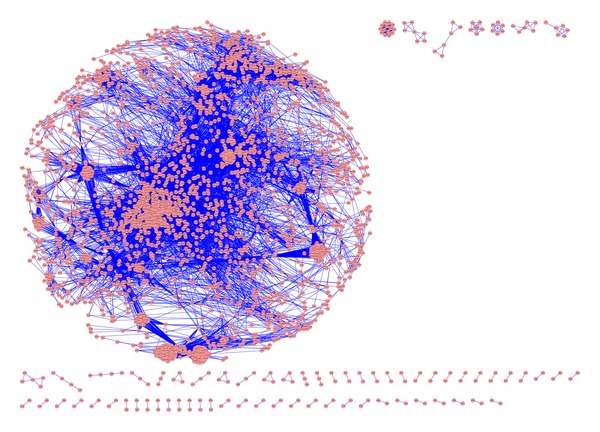
The network consisting of 0.7% Jamu pairs (correlation value above or equal to 0.596).

**Figure 3:**
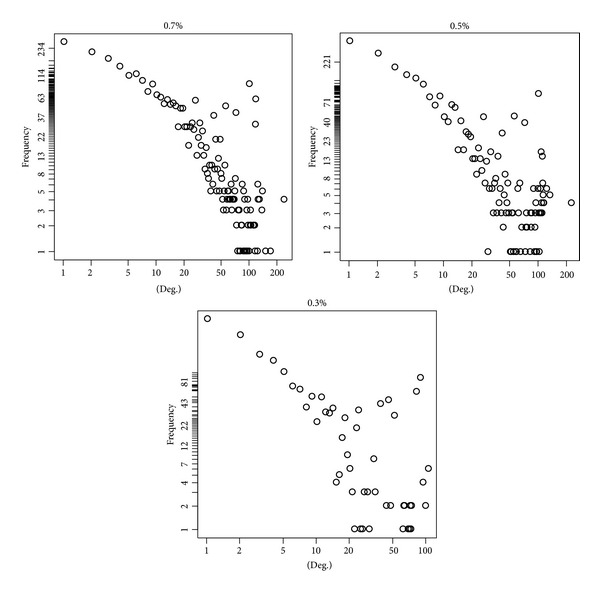
Degree distributions of three Jamu networks roughly follow power law. The *x*-axis corresponds to the log of degree of a node in the Jamu network and the *y*-axis corresponds to the log of the number of Jamu.

After completion of data acquisition process, we calculated the similarity between Jamu pairs using correlation measure. The similarity measures between Jamu pairs were determined based on their ingredients. Corresponding to *K* (3138 in present case) Jamu formulas, there can be maximum (*K* × (*K* − 1)/2) = (3138 × (3137/2)) = 4,921,953 Jamu pairs. We sorted the Jamu pairs based on correlation value using descending order and selected top-*n* (0.7%, 0.5%, and 0.3%) pairs of Jamu formula to create 3 sets of Jamu pairs. The number of Jamu pairs for 0.7%, 0.5%, and 0.3% datasets is 34,454 pairs, 24,610 pairs, and 14,766 pairs and the corresponding minimum correlation values are 0.596, 0.665, and 0.718, respectively. The three datasets of Jamu pairs can be regarded as three undirected networks (step 1 in Figure 1) consisting of 2779, 2496, and 2085 Jamu formulas, respectively (Table 3).  Figure 2 shows visualization of 0.7% Jamu networks using Cytoscape Spring Embedded layout. We verified that the degree distributions of the Jamu networks are somehow close to those of scale-free networks, that is, roughly are of power law type. However, in the high-degree region the power law structure is broken (Figure 3). Nearly accurate relation of power laws between medicinal herbs and the number of formulas utilizing them was observed in Jamu system but not in Kampo (Japanese crude drug system) [4]. The difference of formulas between Jamu and Kampo can be explained by herb selection by medicinal researchers based on the optimization process of selection [4]. Thus, the broken structure of power law corresponding to Jamu networks is associated with the fact that selection of Jamu pairs based on ingredient correlation leads to nonrandom selection. We also constructed random networks according to Erdős-Rényi (ER) model [21], Barabási-Albert (BA) model [22], and Vazquez's Connecting Nearest Neighbor (CNN) model [23] of the same size corresponding to each of the real Jamu network. We used Cytoscape Network Analyzer plugin [24] and R software for analyzing the characteristics of both the Jamu and the random networks.

We determined five statistical indexes, that is, average degree, clustering coefficient, number of connected component, network diameter, and network density of each Jamu network and also of each random network. The clustering coefficient *C*_*n*_ of a node *n* is defined as *C*_*n*_ = 2*e*_*n*_/(*k*_*n*_(*k*_*n*_ − 1)), where *k*_*n*_ is the number of neighbors of *n* and *e*_*n*_ is the number of connected pairs between all neighbors of *n*. The network diameter is the largest distance between any two nodes. If a network is disconnected, its diameter is the maximum of all diameters of its connected components. A network's density is the ratio of the number of edges in the network over the total number of possible edges between all pairs of nodes (which is *n*(*n* − 1)/2, where *n* is the number of vertices, for an undirected graph). The average number of neighbors and the network density are the same for the real and random networks of the same size as it is shown in Table 3. In case of 0.7% and 0.5% real networks, the clustering coefficient is roughly the same and in case of 0.3% the clustering coefficient is somewhat larger. The number of connected components and the diameter of the Jamu networks gradually decrease as the network grows bigger by addition of more nodes and edges.

Very different values corresponding to clustering coefficient, connected component, and network diameter imply that the Jamu networks are quite different from all 3 types of random networks. The differences between Jamu networks and ER random networks are the largest. Random networks constructed based on other two models are also substantially different from Jamu networks. Based on the fact that the random networks constructed based on all three types of models are different from the Jamu networks, it can be concluded that structure of Jamu networks is reasonably biased and thus might contain certain information about plant-disease relations. Specially, much higher value corresponding to clustering coefficient indicates that there are clusters in the networks worthy to be investigated. To extract clusters from the Jamu networks (step 2 in Figure 1) we applied DPClusO network clustering algorithm [14] to generate overlapping clusters based on density and periphery tracking.

### 3.2. Supervised Clustering Based on DPClusO

**Figure 4:**
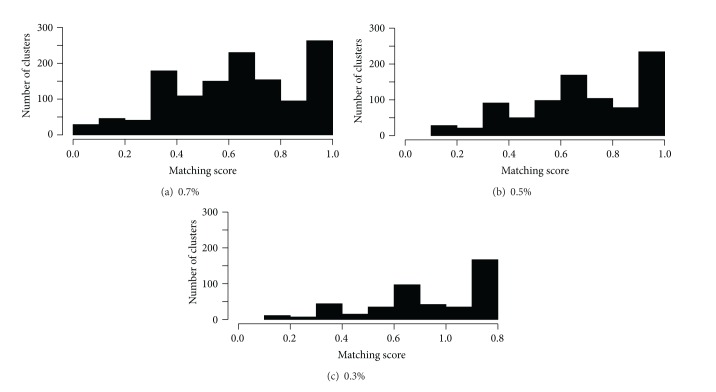
Distribution of clusters based on matching score.

DPClusO is a general-purpose clustering algorithm and useful for finding overlapping cohesive groups in an undirected simple graph for any type of application. It ensures coverage and performs robustly in case of random addition, removal, and rearrangement of edges in protein-protein interaction (PPI) networks [14]. While applying DPClusO, the parameter values of density and cluster property that we used in this experiment are 0.9 and 0.5, respectively [15]. Table 3 shows the summary of clustering result by DPClusO. Because clusters consisting of two Jamu formulas are trivial clusters, for the next steps we only use clusters each of which consists of 3 or more Jamu formulas. The number of total clusters increases along with the larger dataset, although the threshold correlation between Jamu pairs decreases. We evaluated the clustering result using matching score to determine dominant disease for every cluster (step 3 in Figure 1). Matching score of a cluster is the ratio of the highest number of Jamu associated with the same disease to the total number of Jamu in the cluster. Thus matching score is a measure to indicate how strongly a disease is associated to a cluster.  Figure 4 shows the distribution of the clusters with respect to matching score from three datasets. All datasets have the highest frequency of clusters at matching score >0.9 and overall most of the clusters have higher matching score, which means most of the DPClusO generated clusters can be confidently related to a dominant disease. Furthermore the number of clusters with matching score >0.9 is remarkably larger compared to the same in other ranges of matching score in case of the 0.3% dataset (Figure 4(c)). If we compare the ratio of frequency of clusters at matching score >0.9 for every dataset, the 0.3% dataset has the highest ratio with 40.84% (of 453), compared to 29.67% (of 873) and 21.91% (of 1296), in case of 0.5% and 0.7% datasets, respectively. Thus, the most reliable species to disease relations can be predicted at matching score >0.9 corresponding to the clusters generated from 0.3% dataset.

**Figure 5:**
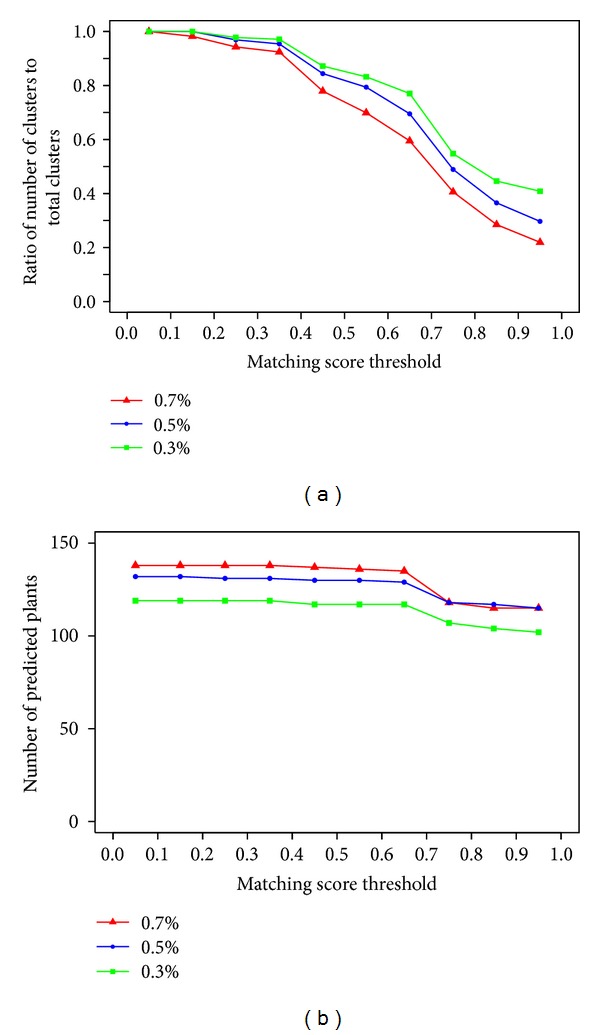
(a) Success rate and (b) number of predicted plants with respect to matching score thresholds.

Figure 5(a) shows the success rate for all 3 datasets with respect to threshold matching scores. Success rate is defined as the ratio of the number of clusters with matching score larger than the threshold to the total number of clusters. As expected it tends to produce lower success rate if we decrease correlation value to create the datasets. However more clusters are generated and more information can be extracted when we lower the threshold correlation value. The success rate increases rapidly as the matching score decreases from 0.9 to 0.6 and after that the slope of increase of success rate decreases. Therefore in this study we empirically decide 0.6 as the threshold matching score to predict plant-disease relations.

### 3.3. Assignment of Plants to Disease

By using DPClusO resulting clusters, we assigned plants to classes of disease. Based on a threshold matching score we assigned dominant disease to a cluster. Then we assign a plant to a cluster by way of analyzing the ingredients of the Jamu formulas belonging to that cluster and determining the highest frequency plant, that is, the plant that is used for maximum number Jamu belonging to that cluster (step 4 in Figure 1). Thus we assign a disease and a plant to each cluster having matching score greater than a threshold. Our hypothesis is that the disease and the plant assigned to the same cluster are related.

**Figure 6:**
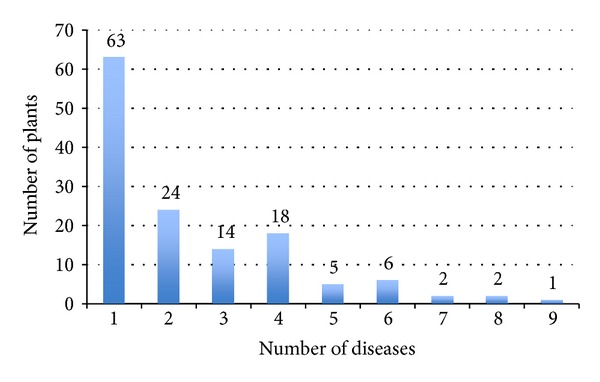
Distribution of 135 plants assigned based on 0.7% dataset with respect to the number of diseases they are assigned to.

The total number of assigned plants depends on matching score value.  Figure 5(b) shows the number of predicted plants that can be assigned to diseases in the context of matching score. With higher matching score value, the number of predicted plants assigned to classes of disease is supposed to remain similar or decrease but the reliability of prediction increases. In Figure 5(b) a sudden change in the number of predicted plants is seen at matching score 0.6 which we consider as empirical threshold in this work. Based on the 0.7% dataset, the largest number of plants (135 plants, Table 4) was assigned to diseases. There are 63 plants assigned to only one class of disease, whereas the other 72 plants are assigned to at least two or more classes of disease (Figure 6).

### 3.4. Evaluation of the Supervised Clustering Based on DPClusO

We used previously published results [6] as gold standard to evaluate our results. The previous study assigned plants to 9 kinds of efficacy whereas we assigned the plants to 18 disease classes (16 from NCBI and 2 additional classes). For the sake of evaluation we got done a mapping of the 18 disease classes to 9 efficacy classes by a professional doctor, which is shown in Table 5.  Table 6 shows the prediction result of plant-disease relations for all 3 datasets, corresponding to clusters with matching score greater than 0.6.  Table 6 also shows corresponding efficacy, the number of assigned plants, number of correctly predicted plants, and true positive rates (TPR), respectively.

We determined TPR corresponding to a disease/efficacy class by calculating the ratio of the number of correct prediction to the number of all predictions. When a disease corresponds to more than one kind of efficacy, the highest TPR can be considered the TPR for the corresponding disease. For all 3 datasets the TPR corresponding to each disease is roughly 90% or more. The 0.3% dataset consists of Jamu pairs with higher correlation values and based on this dataset 117 plants are assigned to 14 disease classes. The 0.7% dataset contains more Jamu pairs and assigned plants to 11 disease classes, one less disease class compared to 0.5% dataset. The two disease classes covered by 0.3% dataset but not covered by 0.5% and 0.7% datasets are the nervous system (D13) and disease of the immune system (D9). The only disease class covered by 0.3% and 0.5% datasets but not covered by 0.7% dataset is mental and behavioural disorders (D18). The larger dataset network tends to have lower coverage of disease classes. The number of Jamu pairs, that is, the number of edges in the network, affect the number of DPClusO resulting clusters and number of Jamu formulas per cluster. As a consequence, for the larger dataset networks, the success rate becomes lower and the coverage of disease classes is lower but prediction of more plant-disease relations can be achieved.

## 4. Conclusions

This paper introduces a novel method called supervised clustering for analyzing big biological data by integrating network clustering and selection of clusters based on supervised learning. In the present work we applied the method for data mining of Jamu formulas accumulated in KNApSAcK database. Jamu networks were constructed based on correlation similarities between Jamu formulas and then network clustering algorithm DPClusO was applied to generate high density Jamu modules. For the analysis of the next steps potential clusters were selected by supervised learning. The successful clusters containing several Jamu related to the same disease might be useful for finding main ingredient plant for that disease and the lower matching score value clusters will be associated with varying plants which might be supporting ingredients. By applying the proposed method important plants from Jamu formulas for every classes of disease were determined. The plant to disease relations predicted by proposed network based method were evaluated in the context of previously published results and were found to produce a TPR of 90%. For the larger dataset networks, success rate and the coverage of disease classes become lower but prediction of more plant-disease relations can be achieved.

## Figures and Tables

**Table 1:** List of diseases using International Classification of Diseases ver. 10 (class of disease IDs correspond to Table 2).

ID	Disease	Class of disease
1	Abdominal pain	3
2	Abdominal pain, diarrhea	3
3	Acne	16
4	Acne, skin problems (cosmetics)	16
5	Amenorrhoea, dysmenorrhea	6
6	Amenorrhoea, irregular menstruation	6
7	Anaemia	1
8	Appendicitis, urinary tract infection, tonsillitis	3
9	Arthralgia	11
10	Arthralgia, arthritis	11
11	Asthma	15
12	Benign prostatic hyperplasia (Bph)	10
13	Breast disorder	6
14	Bromhidrosis	16
15	Bronchitis	15
16	Cancer	2
17	Cancer pain	2
18	Cancer, inflammation	2
19	Colic abdomen, bloating (in infant)	3
20	Common cold	15
21	Common cold, dyspepsia, insect bites	15, 3, 16
22	Common cold, influenza	15
23	Cough	15
24	Degenerative disease	14
25	Dermatitis, urticaria, erythema	16
26	Diabetes	14
27	Diabetic gangrene	16
28	Diarrhea	3
29	Diarrhea, abdominal pain	3
30	Diseases of the eye	5
31	Disorders in pregnancy	6
32	Dysmenorrhea	6
33	Dysmenorrhea, irregular menstruation	6
34	Dysmenorrhea, menstrual syndrome	6
35	Dyspepsia	3
36	Dyspnoea	15
37	Dyspnoea, cough, orthopnoea	15
38	Fatigue	11
39	Fatigue, anaemia, loss appetite	1
40	Fatigue, lack of sexual function	6
41	Fatigue, low back pain	11
42	Fatigue, myalgia, arthralgia	11
43	Fatigue, osteoarthritis	11
44	Fertility problem	6, 10
45	Fever	0
46	Gastritis, gastric ulcer	3
47	Haemorrhoids	1
48	Headache	13
49	Heart diseases	8
50	Heartburn	3, 8
51	Hepatitis, other diseases of liver	3
52	Hypercholesterolaemia	14
53	Hypertension	8
54	Hypertension, diabetes	14
55	Hypertension, hypercholesterolaemia	14
56	Hyperuricemia	1
57	Immunodefficiency	9
58	Indigestion (K.30)	3
59	Indigestion, lose appetite	3
60	Infertility	6, 10
61	Irregular menstruation, menstruation syndrome	6
62	Kidney diseases	17
63	Lactation problems	6
64	Leukorrhoea (Vaginalis)	6
65	Leukorrhoea (Vaginalis), dysmenorrhoea	6
66	Lose appetite	3
67	Lose appetite, underweight	14
68	Low back pain, myalgia, arthralgia	11
69	Low back pain, myalgia, constipation	11
70	Low back pain, urinary tract infection	17
71	Lung diseases	15
72	Malaise and Fatigue	11
73	Malaise and Fatigue, Constipation	11
74	Malaise and Fatigue, Fertility Problems	10, 11
75	Malaise and Fatigue, Low Back Pain	11
76	Malaise and Fatigue, Sexual Dysfunction	11, 6, 10
77	Malaise and Fatigue, Skin Problems (Cosmetics)	16
78	Malaria, anaemia	1
79	Meno-metrorrhagia	6
80	Menopausal syndrome	6
81	Menopause/menstrual syndrome, leukorrhoea (vaginalis)	6
82	Menstrual syndrome	6
83	Menstrual syndrome, fatigue	6
84	Migraine	13
85	Mood disorder	18
86	Myalgia, arthralgia	11
87	Nausea/vomiting of pregnancy	6
88	Osteoarthritis	11
89	Osteoarthritis, fatigue	11
90	Overweight, obesity	14
91	Paralysis	13
92	Post partum syndrome	6
93	Prevent from overweight	14
94	Respiratory infection due to smoking	15
95	Respiratory tract infection	15
96	Rheumatoid arthritis, gout	11
97	Secondary amenorrhea	6
98	Secondary amenorrhea, irregular menstruation	6
99	Sexual dysfunction, fatigue	6, 10
100	Skin diseases	16
101	Skin problems (cosmetics)	16
102	Sleeping and Mood Disorders	18
103	Sleeping disorders	18
104	Stomatitis	3
105	Stomatitis, gingivitis, tonsilitis	3
106	Stone in kidney (N20.0)	17
107	Stone in kidney (N20.0), urinary bladder stone (N21.0)	17
108	Tonsilitis	4
109	Tonsilofaringitis	4
110	Toothache	13
111	Typhoid, dyspepsia	3
112	Ulcer of anus and rectum	3
113	Underweight, lose appetite	3
114	Urinary tract infection (urethritis)	17
115	Vaginal discharges	6
116	Vaginal diseases	6

**Table 2:** Distribution of Jamu formulas according to 18 classes of disease (classes of diseases are determined by NCBI in ID1 to ID16 and by the present study in ID17 and ID18 represented by asterisks in Ref. columns).

ID	Class of disease (NCBI)	Ref.	Number of Jamu	Percentage
1	Blood and lymph diseases	NCBI	201	6.41
2	Cancers	NCBI	32	1.02
3	The digestive system	NCBI	457	14.56
4	Ear, nose, and throat	NCBI	2	0.06
5	Diseases of the eye	NCBI	1	0.03
6	Female-specific diseases	NCBI	382	12.17
7	Glands and hormones	NCBI	0	—
8	The heart and blood vessels	NCBI	57	1.82
9	Diseases of the immune system	NCBI	22	0.70
10	Male-specific diseases	NCBI	17	0.54
11	Muscle and bone	NCBI	649	20.68
12	Neonatal diseases	NCBI	0	—
13	The nervous system	NCBI	32	1.02
14	Nutritional and metabolic diseases	NCBI	576	18.36
15	Respiratory diseases	NCBI	313	9.97
16	Skin and connective tissue	NCBI	163	5.19
17	The urinary system	∗	90	2.87
18	Mental and behavioral disorders	∗	21	0.67

	The number of Jamu classified into multiple disease classes		119	3.79
	The number of Jamu unclassified		4	0.13

	Total Jamu formulas		3138	100.00

**Table 3:** Statistics of three datasets.

	Parameters	0.7%	0.5%	0.3%
Network statistics	Total pairs	34,454	24,610	14,766
	Minimum correlation	0.596	0.665	0.718
	Number of Jamu formulas	2,779	2,496	2,085
	Average degree	24.8	19.7	14.2
	(Random network: ER)	(24.8 ± 0.0)	(19.7 ± 0.0)	(14.2 ± 0.0)
	(Random network: BA)	(24.7 ± 0.1)	(19.7 ± 0.1)	(14.1 ± 0.1)
	(Random network: CNN)	(24.7 ± 0.4)	(19.7 ± 0.4)	(14.0 ± 0.4)
	Clustering coefficient	0.521	0.520	0.540
	(Random network: ER)	(0.009 ± 0.000)	(0.008 ± 0.000)	(0.007 ± 0.000)
	(Random network: BA)	(0.030 ± 0.001)	(0.028 ± 0.001)	(0.026 ± 0.001)
	(Random network: CNN)	(0.246 ± 0.008)	(0.239 ± 0.008)	(0.233 ± 0.010)
	Number of connected components	69	119	254
	(Random networks: ER, BA, CNN)	(1)	(1)	(1)
	Network diameter	15	17	20
	(Random network: ER)	(4.0 ± 0.0)	(4.0 ± 0.0)	(5.0 ± 0.0)
	(Random network: BA)	(10.8 ± 0.8)	(11.2 ± 1.5)	(10.8 ± 0.9)
	(Random network: CNN)	(14.6 ± 1.9)	(14.1 ± 1.4)	(14.7 ± 1.3)
	Network density	0.008	0.008	0.007
	(Random network: ER)	(0.009 ± 0.000)	(0.008 ± 0.000)	(0.007 ± 0.000)
	(Random network: BA)	(0.009 ± 0.000)	(0.008 ± 0.000)	(0.007 ± 0.000)
	(Random network: CNN)	(0.009 ± 0.000)	(0.008 ± 0.000)	(0.007 ± 0.000)

DPClusO	Total number of clusters	1,746	1,411	938
	Number of clusters with more than 2 Jamu	1,296	873	453
	(%)	(74.2)	(61.9)	(48.3)
	Number of Jamu formulas in the biggest cluster	118	104	89

**Table 4:** List of plants assigned to each disease.

Number	Plants name	Hit-miss status	
*A. Disease: blood and lymph diseases *			
1	*Tamarindus indica *	*Hit *	∗
2	*Allium sativum *	*Hit *	∗
3	*Tinospora tuberculata *	*Hit *	∗
4	*Piper retrofractum *	*Hit *	
5	*Syzygium aromaticum *	*Hit *	∗
6	*Bupleurum falcatum *	*Hit *	
7	*Graptophyllum pictum *	*Hit *	
8	*Plantago major *	*Hit *	
9	*Zingiber officinale *	*Hit *	∗
10	*Cinnamomum burmannii *	*Hit *	∗
11	*Soya max *	*Miss *	∗
12	*Kaempferia galanga *	*Hit *	
13	*Curcuma longa *	*Hit *	∗
14	*Piper nigrum *	*Hit *	
15	*Zingiber aromaticum *	*Hit *	∗
16	*Phyllanthus urinaria *	*Hit *	∗
17	*Oryza sativa *	*Hit *	
18	*Myristica fragrans *	*Hit *	∗
19	*Alstonia scholaris *	*Hit *	∗
20	*Syzygium polyanthum *	*Miss *	
21	*Andrographis paniculata *	*Hit *	∗
22	*Sida rhombifolia *	*Miss *	
23	*Cyperus rotundus *	*Hit *	
24	*Sonchus arvensis *	*Miss *	
25	*Curcuma aeruginosa *	*Hit *	∗
26	*Curcuma xanthorrhiza *	*Hit *	

*B. Disease: cancers *			
1	*Catharanthus roseus *	*Hit *	

*C. Disease: the digestive system *			
1	*Foeniculum vulgare *	*Hit *	
2	*Glycyrrhiza uralensis *	*Hit *	∗
3	*Imperata cylindrica *	*Hit *	
4	*Zingiber purpureum *	*Hit *	∗
5	*Physalis peruviana *	*Hit *	
6	*Punica granatum *	*Hit *	∗
7	*Echinacea purpurea *	*Hit *	
8	*Zingiber officinale *	*Hit *	∗
9	*Psidium guajava *	*Hit *	
10	*Baeckea frutescens *	*Hit *	∗
11	*Amomum compactum *	*Hit *	
12	*Cinnamomum burmannii *	*Hit *	∗
13	*Melaleuca leucadendra *	*Hit *	
14	*Caesalpinia sappan *	*Hit *	∗
15	*Parkia roxburghii *	*Hit *	
16	*Rheum tanguticum *	*Hit *	
17	*Kaempferia galanga *	*Hit *	
18	*Coriandrum sativum *	*Hit *	
19	*Curcuma longa *	*Hit *	
20	*Zingiber aromaticum *	*Hit *	
21	*Phyllanthus urinaria *	*Hit *	
22	*Myristica fragrans *	*Hit *	
23	*Hydrocotyle asiatica *	*Hit *	∗
24	*Carica papaya *	*Hit *	
25	*Mentha arvensis *	*Hit *	
26	*Lepiniopsis ternatensis *	*Hit *	
27	*Helicteres isora *	*Hit *	
28	*Andrographis paniculata *	*Hit *	
29	*Symplocos odoratissima *	*Hit *	
30	*Schisandra chinensis *	*Hit *	
31	*Blumea balsamifera *	*Hit *	
32	*Silybum marianum *	*Hit *	∗
33	*Cinnamomum sintoc *	*Hit *	
34	*Elephantopus scaber *	*Hit *	
35	*Curcuma aeruginosa *	*Hit *	
36	*Kaempferia pandurata *	*Hit *	
37	*Curcuma xanthorrhiza *	*Hit *	
38	*Curcuma mangga *	*Hit *	∗
39	*Curcuma zedoaria *	*Hit *	
40	*Daucus carota *	*Hit *	∗
41	*Matricaria chamomilla *	*Hit *	∗
42	*Cymbopogon nardus *	*Hit *	∗

*D. Disease: female-specific diseases *			
1	*Foeniculum vulgare *	*Hit *	
2	*Imperata cylindrica *	*Hit *	
3	*Tamarindus indica *	*Hit *	
4	*Pluchea indica *	*Hit *	∗
5	*Piper retrofractum *	*Hit *	
6	*Punica granatum *	*Hit *	
7	*Uncaria rhynchophylla *	*Hit *	
8	*Zingiber officinale *	*Hit *	
9	*Guazuma ulmifolia *	*Hit *	∗
10	*Nigella sativa *	*Hit *	
11	*Terminalia bellirica *	*Hit *	
12	*Baeckea frutescens *	*Hit *	
13	*Phaseolus radiatus *	*Hit *	
14	*Amomum compactum *	*Hit *	∗
15	*Sauropus androgynus *	*Hit *	
16	*Usnea misaminensis *	*Hit *	
17	*Cinnamomum burmannii *	*Hit *	
18	*Melaleuca leucadendra *	*Hit *	
19	*Parameria laevigata *	*Hit *	
20	*Parkia roxburghii *	*Hit *	
21	*Piper cubeba *	*Hit *	
22	*Kaempferia galanga *	*Hit *	
23	*Coriandrum sativum *	*Hit *	
24	*Kaempferia angustifolia *	*Hit *	
25	*Curcuma longa *	*Hit *	
26	*Zingiber aromaticum *	*Hit *	
27	*Languas galanga *	*Hit *	
28	*Galla lusitania *	*Hit *	
29	*Quercus lusitanica *	*Hit *	
30	*Hydrocotyle asiatica *	*Hit *	
31	*Areca catechu *	*Hit *	
32	*Lepiniopsis ternatensis *	*Hit *	
33	*Helicteres isora *	*Hit *	∗
34	*Piper betle *	*Hit *	
35	*Elephantopus scaber *	*Hit *	∗
36	*Kaempferia pandurata *	*Hit *	
37	*Curcuma xanthorrhiza *	*Hit *	
38	*Sesbania grandiflora *	*Hit *	

*E. Disease: the heart and blood vessels *			
1	*Allium sativum *	*Hit *	
2	*Curcuma longa *	*Hit *	∗
3	*Morinda citrifolia *	*Hit *	∗
4	*Homalomena occulta *	*Hit *	∗
5	*Hydrocotyle asiatica *	*Hit *	
6	*Alstonia scholaris *	*Hit *	∗
7	*Syzygium polyanthum *	*Miss *	∗
8	*Andrographis paniculata *	*Hit *	∗
9	*Apium graveolens *	*Miss *	
10	*Imperata cylindrica *	*Hit *	

*F. Disease: male-specific diseases *			
1	*Cucurbita pepo *	*Miss *	
2	*Serenoa repens *	*Miss *	
3	*Baeckea frutescens *	*Hit *	
4	*Phaseolus radiatus *	*Hit *	
5	*Curcuma longa *	*Hit *	
6	*Elephantopus scaber *	*Hit *	

*G. Disease: muscle and bone *			
1	*Foeniculum vulgare *	*Hit *	
2	*Clausena anisum-olens *	*Hit *	∗
3	*Zingiber purpureum *	*Hit *	
4	*Allium sativum *	*Hit *	
5	*Strychnos ligustrina *	*Hit *	
6	*Tinospora tuberculata *	*Hit *	∗
7	*Piper retrofractum *	*Hit *	
8	*Syzygium aromaticum *	*Hit *	
9	*Cola nitida *	*Hit *	∗
10	*Ginkgo biloba *	*Hit *	∗
11	*Panax ginseng *	*Hit *	
12	*Equisetum debile *	*Hit *	∗
13	*Zingiber officinale *	*Hit *	
14	*Ganoderma lucidum *	*Hit *	
15	*Nigella sativa *	*Hit *	
16	*Terminalia bellirica *	*Hit *	∗
17	*Baeckea frutescens *	*Hit *	∗
18	*Amomum compactum *	*Hit *	
19	*Cinnamomum burmannii *	*Hit *	
20	*Melaleuca leucadendra *	*Hit *	
21	*Parameria laevigata *	*Hit *	∗
22	*Psophocarpus tetragonolobus *	*Hit *	∗
23	*Parkia roxburghii *	*Hit *	
24	*Piper cubeba *	*Hit *	∗
25	*Kaempferia galanga *	*Hit *	
26	*Coriandrum sativum *	*Hit *	
27	*Cola acuminata *	*Hit *	
28	*Coffea arabica *	*Hit *	
29	*Orthosiphon stamineus *	*Hit *	
30	*Curcuma longa *	*Hit *	
31	*Piper nigrum *	*Hit *	
32	*Alpinia galanga *	*Hit *	
33	*Vitex trifolia *	*Hit *	
34	*Zingiber amaricans *	*Hit *	∗
35	*Zingiber zerumbet *	*Hit *	
36	*Zingiber aromaticum *	*Hit *	
37	*Languas galanga *	*Hit *	
38	*Massoia aromatica *	*Hit *	
39	*Morinda citrifolia *	*Hit *	
40	*Carum copticum *	*Hit *	∗
41	*Panax pseudoginseng *	*Hit *	∗
42	*Oryza sativa *	*Hit *	
43	*Myristica fragrans *	*Hit *	
44	*Pandanus amaryllifolius *	*Hit *	
45	*Eurycoma longifolia *	*Hit *	
46	*Hydrocotyle asiatica *	*Hit *	
47	*Areca catechu *	*Hit *	∗
48	*Mentha arvensis *	*Hit *	∗
49	*Lepiniopsis ternatensis *	*Hit *	
50	*Pimpinella pruatjan *	*Hit *	
51	*Andrographis paniculata *	*Hit *	
52	*Blumea balsamifera *	*Hit *	
53	*Cymbopogon nardus *	*Hit *	
54	*Sida rhombifolia *	*Hit *	
55	*Cinnamomum sintoc *	*Hit *	
56	*Piper betle *	*Hit *	∗
57	*Talinum paniculatum *	*Hit *	
58	*Elephantopus scaber *	*Hit *	
59	*Cyperus rotundus *	*Hit *	
60	*Curcuma aeruginosa *	*Hit *	
61	*Kaempferia pandurata *	*Hit *	∗
62	*Curcuma xanthorrhiza *	*Hit *	
63	*Tribulus terrestris *	*Hit *	
64	*Corydalis yanhusuo *	*Hit *	
65	*Pausinystalia yohimbe *	*Hit *	

*H. Disease: nutritional and metabolic diseases *			
1	*Foeniculum vulgare *	*Hit *	
2	*Glycyrrhiza uralensis *	*Hit *	
3	*Zingiber purpureum *	*Hit *	
4	*Allium sativum *	*Hit *	
5	*Tinospora tuberculata *	*Hit *	
6	*Pandanus conoideus *	*Hit *	
7	*Syzygium aromaticum *	*Hit *	
8	*Punica granatum *	*Hit *	
9	*Zingiber officinale *	*Hit *	
10	*Guazuma ulmifolia *	*Hit *	
11	*Nigella sativa *	*Hit *	
12	*Amomum compactum *	*Hit *	∗
13	*Cinnamomum burmannii *	*Hit *	
14	*Parameria laevigata *	*Hit *	
15	*Caesalpinia sappan *	*Hit *	
16	*Soya max *	*Hit *	∗
17	*Cocos nucifera *	*Hit *	
18	*Rheum tanguticum *	*Hit *	
19	*Piper cubeba *	*Hit *	∗
20	*Murraya paniculata *	*Hit *	
21	*Kaempferia galanga *	*Hit *	∗
22	*Coffea arabica *	*Hit *	∗
23	*Orthosiphon stamineus *	*Hit *	
24	*Curcuma longa *	*Hit *	
25	*Piper nigrum *	*Hit *	∗
26	*Zingiber aromaticum *	*Hit *	
27	*Aloe vera *	*Hit *	
28	*Phaleria papuana *	*Hit *	
29	*Galla lusitania *	*Hit *	
30	*Quercus lusitanica *	*Hit *	
31	*Morinda citrifolia *	*Hit *	
32	*Myristica fragrans *	*Hit *	∗
33	*Momordica charantia *	*Hit *	
34	*Areca catechu *	*Hit *	
35	*Lepiniopsis ternatensis *	*Hit *	
36	*Alstonia scholaris *	*Hit *	
37	*Hibiscus sabdariffa *	*Hit *	
38	*Laminaria japonica *	*Hit *	
39	*Syzygium polyanthum *	*Hit *	
40	*Andrographis paniculata *	*Hit *	
41	*Sindora sumatrana *	*Hit *	∗
42	*Cassia angustifolia *	*Hit *	
43	*Woodfordia floribunda *	*Hit *	
44	*Piper betle *	*Hit *	
45	*Spirulina *	*Hit *	
46	*Stevia rebaudiana *	*Hit *	
47	*Theae sinensis *	*Hit *	
48	*Sonchus arvensis *	*Hit *	
49	*Curcuma heyneana *	*Hit *	
50	*Curcuma aeruginosa *	*Hit *	
51	*Kaempferia pandurata *	*Hit *	∗
52	*Curcuma xanthorrhiza *	*Hit *	
53	*Curcuma zedoaria *	*Hit *	∗
54	*Olea europaea *	*Hit *	

*I. Disease respiratory diseases *			
1	*Foeniculum vulgare *	*Hit *	
2	*Clausena anisum-olens *	*Hit *	
3	*Glycyrrhiza uralensis *	*Hit *	
4	*Zingiber purpureum *	*Hit *	
5	*Piper retrofractum *	*Hit *	∗
6	*Syzygium aromaticum *	*Hit *	
7	*Gaultheria punctata *	*Hit *	
8	*Panax ginseng *	*Hit *	
9	*Equisetum debile *	*Hit *	∗
10	*Zingiber officinale *	*Hit *	
11	*Citrus aurantium *	*Hit *	∗
12	*Nigella sativa *	*Hit *	∗
13	*Amomum compactum *	*Hit *	
14	*Cinnamomum burmannii *	*Hit *	
15	*Melaleuca leucadendra *	*Hit *	
16	*Parkia roxburghii *	*Hit *	
17	*Cocos nucifera *	*Hit *	
18	*Piper cubeba *	*Hit *	
19	*Kaempferia galanga *	*Hit *	
20	*Coriandrum sativum *	*Hit *	
21	*Curcuma longa *	*Hit *	
22	*Piper nigrum *	*Hit *	
23	*Zingiber aromaticum *	*Hit *	
24	*Languas galanga *	*Hit *	
25	*Mentha piperita *	*Hit *	
26	*Oryza sativa *	*Hit *	∗
27	*Myristica fragrans *	*Hit *	
28	*Pandanus amaryllifolius *	*Hit *	∗
29	*Hydrocotyle asiatica *	*Hit *	∗
30	*Mentha arvensis *	*Hit *	
31	*Lepiniopsis ternatensis *	*Hit *	
32	*Helicteres isora *	*Hit *	
33	*Blumea balsamifera *	*Hit *	
34	*Cymbopogon nardus *	*Hit *	
35	*Piper betle *	*Hit *	
36	*Curcuma xanthorrhiza *	*Hit *	
37	*Salix alba *	*Hit *	∗
38	*Matricaria chamomilla *	*Miss *	∗

*J. Disease: skin and connective tissue *			
1	*Strychnos ligustrina *	*Hit *	
2	*Merremia mammosa *	*Hit *	∗
3	*Piper retrofractum *	*Hit *	∗
4	*Santalum album *	*Hit *	
5	*Zingiber officinale *	*Hit *	∗
6	*Citrus aurantium *	*Hit *	
7	*Citrus hystrix *	*Hit *	
8	*Cassia siamea *	*Hit *	
9	*Cocos nucifera *	*Hit *	
10	*Trigonella foenum-graecum *	*Hit *	
11	*Orthosiphon stamineus *	*Hit *	
12	*Curcuma longa *	*Hit *	
13	*Vetiveria zizanioides *	*Hit *	
14	*Aloe vera *	*Hit *	
15	*Rosa chinensis *	*Hit *	
16	*Jasminum sambac *	*Hit *	
17	*Phyllanthus urinaria *	*Hit *	
18	*Mentha piperita *	*Hit *	
19	*Oryza sativa *	*Hit *	
20	*Myristica fragrans *	*Hit *	∗
21	*Hydrocotyle asiatica *	*Hit *	
22	*Lepiniopsis ternatensis *	*Hit *	
23	*Alstonia scholaris *	*Hit *	
24	*Andrographis paniculata *	*Hit *	
25	*Cymbopogon nardus *	*Hit *	
26	*Piper betle *	*Hit *	
27	*Theae sinensis *	*Hit *	
28	*Curcuma heyneana *	*Hit *	
29	*Kaempferia pandurata *	*Hit *	∗
30	*Curcuma xanthorrhiza *	*Hit *	
31	*Melaleuca leucadendra *	*Hit *	
32	*Matricaria chamomilla *	*Miss *	∗

*K. Disease: the urinary system *			
1	*Foeniculum vulgare *	*Hit *	∗
2	*Imperata cylindrica *	*Hit *	∗
3	*Strychnos ligustrina *	*Hit *	∗
4	*Plantago major *	*Hit *	
5	*Zingiber officinale *	*Hit *	∗
6	*Cinnamomum burmannii *	*Hit *	∗
7	*Strobilanthes crispus *	*Hit *	
8	*Kaempferia galanga *	*Hit *	∗
9	*Orthosiphon stamineus *	*Hit *	
10	*Phyllanthus urinaria *	*Hit *	
11	*Blumea balsamifera *	*Hit *	∗
12	*Sonchus arvensis *	*Hit *	
13	*Curcuma xanthorrhiza *	*Hit *	

*indicates that plant will not assigned if we use matching score >0.7.

**Table 5:** Relation between disease classes in NCBI and efficacy classes reported by Afendi et al. [6].

Class of disease	Ref.	Efficacy class
**D1** Blood and lymph diseases	NCBI	**E7** Pain/inflammation (PIN)
**D2** Cancers	NCBI	**E7** Pain/inflammation (PIN)
**D3** The digestive system	NCBI	**E4** Gastrointestinal disorders (GST)
		**E7** Pain/inflammation (PIN)
**D4** Ear, nose, and throat	NCBI	**E7** Pain/inflammation (PIN)
**D5** Diseases of the eye	NCBI	**E7** Pain/inflammation (PIN)
**D6** Female-specific diseases	NCBI	**E5** Female reproductive organ problems (FML)
**D7** Glands and hormones	NCBI	**E7** Pain/inflammation (PIN)
**D8** The heart and blood vessels	NCBI	**E7** Pain/inflammation (PIN)
**D9** Diseases of the immune system	NCBI	**E7** Pain/inflammation (PIN)
**D10** Male-specific diseases	NCBI	**E6** Musculoskeletal and connective tissue disorders (MSC)
**D11** Muscle and bone	NCBI	**E6** Musculoskeletal and connective tissue disorders (MSC)
**D12** Neonatal diseases	NCBI	**E7** Pain/inflammation (PIN)
**D13** The nervous system	NCBI	**E7** Pain/inflammation (PIN)
**D14** Nutritional and metabolic diseases	NCBI	**E2** Disorders of appetite (DOA)
		**E4** Gastrointestinal disorders (GST)
**D15** Respiratory diseases	NCBI	**E8** Respiratory disease (RSP)
		**E7** Pain/inflammation (PIN)
**D16** Skin and connective tissue	NCBI	**E9** Wounds and skin infections (WND)
**D17** The urinary system	∗	**E1** Urinary related problems (URI)
**D18** Mental and behavioural disorders	∗	**E3** Disorders of mood and behavior (DMB)

**Table 6:** The prediction result of plant-disease relations using matching score >0.6.

Class of disease	Corresponding efficacy	0.7% dataset			0.5% dataset			0.3% dataset		
		Number of assigned plants	Correct prediction	True positive rate	Number of assigned plants	Correct prediction	True positive rate	Number of assigned plants	Correct prediction	True positive rate
D1	E7	26	22	0.85	24	20	0.83	24	20	0.83
D2	E7	1	1	1.00	5	5	1.00	1	1	1.00
D3	E4	42	42	1.00	33	33	1.00	28	28	1.00
	E7		38	0.90		30	0.91		25	0.89
D4	E7	0	0	—	0	0	—	0	0	—
D5	E7	0	0	—	0	0	—	0	0	—
D6	E5	38	38	1.00	37	37	1.00	32	32	1.00
D7	E7	0	0	—	0	0	—	0	0	—
D8	E7	10	8	0.80	8	7	0.88	6	5	0.83
D9	E7	0	0	—	0	0	—	1	1	1.00
D10	E6	6	4	0.67	2	0	—	3	1	0.33
D11	E6	65	65	1.00	71	71	1.00	60	60	1.00
D12	E7	0	0	—	0	0	—	0	0	—
D13	E7	0	0	—	0	0	—	5	5	1.00
D14	E2	54	44	0.81	45	36	0.80	35	26	0.74
	E4		54	1.00		45	1.00		35	1.00
D15	E7	38	37	0.97	34	34	1.00	33	33	1.00
	E8		31	0.82		30	0.88		29	0.88
D16	E9	32	31	0.97	32	32	1.00	27	27	1.00
D17	E1	13	13	1.00	9	9	1.00	8	8	1.00
D18	E3	0	0	—	5	5	1.00	4	4	1.00

Total assigned plants		135			129			117		
